# A Caspase-3 Reporter for Fluorescence Lifetime Imaging of Single-Cell Apoptosis

**DOI:** 10.3390/cells7060057

**Published:** 2018-06-13

**Authors:** Johanna M. Buschhaus, Brock Humphries, Kathryn E. Luker, Gary D. Luker

**Affiliations:** 1 Center for Molecular Imaging, Department of Radiology, University of Michigan, Ann Arbor, MI 48190, USA; jbuschha@umich.edu (J.M.B.); brhu@umich.edu (B.H.); kluker@umich.edu (K.E.L.); 2 Department of Biomedical Engineering, University of Michigan, Ann Arbor, MI 48190, USA; 3 Department of Microbiology and Immunology, University of Michigan, Ann Arbor, MI 48190, USA

**Keywords:** apoptosis, breast cancer, caspase-3, fluorescence lifetime imaging, Förster resonance energy transfer

## Abstract

Fluorescence lifetime imaging (FLIM) is a powerful imaging modality used to gather fluorescent reporter data independent of intracellular reporter intensity or imaging depth. We applied this technique to image real-time activation of a reporter for the proteolytic enzyme, caspase-3, in response to apoptotic cell death. This caspase-3 reporter activity provides valuable insight into cancer cell responsiveness to therapy and overall viability at a single-cell scale. Cleavage of a aspartate-glutamate-valine-aspartate (DEVD) linkage sequence alters Förster resonance energy transfer (FRET) within the reporter, affecting its lifetime. Cellular apoptosis was quantified in multiple environments ranging from 2D flat and 3D spheroid cell culture systems to in vivo murine mammary tumor xenografts. We evaluated cell-by-cell apoptotic responses to multiple pharmacological and genetic methods of interest involved in cancer cell death. Within this article, we describe methods for measuring caspase-3 activation at single-cell resolution in various complex environments using FLIM.

## 1. Introduction

Apoptosis, or programmed cell death, is a tightly regulated cell suicide program that is critical during normal development and in cancer treatment [1]. Apoptosis involves a signaling cascade that, ultimately, activates a family of proteases known as caspases. In response to various stimuli, apoptosis begins with the activation of “initiator” caspases, which cleave and activate “executioner” caspases (caspase-3, -6, and/or -7) [1,2,3]. Upon activation, these “executioner” caspases cleave various structural and repair proteins at the aspartate-glutamate-valine-aspartate (DEVD) amino acid sequence, resulting in apoptotic cell death [2]. Current cytotoxic anticancer therapies commonly induce apoptosis of malignant cells. However, cancer cells can employ one or multiple mechanisms to evade apoptosis.

Recent advances in fluorescence microscopy and fluorescent probe development have increased our understanding of cellular biology and cancer progression. Förster resonance energy transfer (FRET) is one such technique that has greatly contributed to our understanding of protein interactions and signaling within a cell. FRET is a distance-dependent physical phenomenon by which energy is transferred from one excited fluorophore (also known as the donor) to a nearby acceptor, in this case, another nearby fluorophore [3]. For FRET to occur between two fluorescent proteins, the emission spectrum of the donor molecule must overlap with the excitation spectrum of the acceptor. Since this reaction is distance-dependent, FRET can be used to determine interactions of proteins or protein domains of up to a distance of 10–100 Å [4]. FRET is typically measured ratiometrically, using the relative fluorescence intensities of the excited donor and acceptor proteins. Efficient FRET is defined as a reduction of donor fluorescence relative to acceptor fluorescence. However, intensity-based FRET can be distorted by relative concentrations of donor and acceptor molecules, sample movement, excitation fluctuation, as well as the wavelength-dependent differences in attenuation and scattering of light in tissues [5,6,7]. 

To overcome deficiencies common to ratiometric FRET, we, and others, have employed fluorescence lifetime imaging microscopy (FLIM) to quantify FRET. FLIM measures changes in fluorescence lifetime based on phase delay and the modulation ratio of light emitted from a fluorescent molecule relative to the excitation light [8]. The lifetime of a fluorophore is the measure of time an excited fluorophore stays in an excited state before returning to its ground state [9]. FRET interactions shorten the fluorescence lifetime of a donor fluorophore. FLIM offers several advantages over other imaging techniques for quantifying FRET. Primarily, unlike intensity-based ratiometric quantification, the fluorescence lifetime of a molecule is independent of the probe concentration or tissue depth. Therefore, this technique is better suited for 3D and in vivo models. FLIM also helps eliminate problems of bleed-through fluorescence from the donor molecule or direct excitation of the acceptor.

Here, we used FLIM to monitor a caspase-3 FRET imaging reporter to quantify apoptosis in in vitro and in vivo models [10,11,12,13,14]. The specifics of the FRET pair, LSS-mOrabge and mKate2, have been previously described [8]. The caspase-3 reporter is engineered to link LSS-mOrange (donor) and mKate2 (acceptor) fluorescent molecules through the canonical DEVD sequence recognized by the caspase-3 enzyme. LSS-mOrange represents the ideal donor as the long Stokes shift can easily separate the excitation of LSS-mOrange from mKate2, while still providing a large spectral overlap for FRET to occur. In cells that do not activate caspase-3, the reporter remains intact and allows FRET to occur upon excitation of LSS-mOrange, reducing its lifetime. Cells that activate caspase-3 cleave the DEVD sequence that links LSS-mOrange and mKate2, causing the two fluorescent proteins to separate and the lifetime of LSS-mOrange increases. Therefore, cells that undergo apoptosis will display reduced FRET, which correlates to a longer lifetime of LSS-mOrange.

In the following protocol, we describe in detail the necessary steps to measure apoptosis via FLIM-FRET in different experimental models and in response to various treatments. The first protocol explains how to generate cells that contain the LSSmOrange-DEVD-mKate2 apoptosis imaging reporter, as well as the appropriate control cells. We use these cells to describe how to detect apoptosis levels in 2D cell culture, 3D spheroids, and in vivo mouse models. We describe useful genetic perturbations, as well as pharmaceutical drugs, as a means to alter apoptosis. We also list selected methods to validate imaging data acquired for caspase-3 activation and apoptosis. Overall, the imaging methods described here offer new opportunities to investigate cell apoptosis at the single cell level in both 2D and 3D systems, which can enhance our understanding of tumor heterogeneity and drug resistance.

## 2. Protocol 1: Generating and Maintaining Stable Cell Lines

In Protocol 1, we describe how to transduce HEK-293T human embryonic kidney and MDA-MB-231 metastatic breast cancer cell lines to stably express either the control reporter, LSS-mOrange, or the caspase-3 apoptosis reporter, LSS-mOrange-DEVD-mKate2. Other cell lines may be used for constitutive expression and experiments at investigators’ discretions. First, we outline the creation of the lentiviral vector, LSS-mOrange pLVX IRES blasticidin, and the PiggyBac transposon vector, LSS-mOrange-DEVD-mKate2. Stably-expressing cell lines are created by inserting the vectors into cells. Finally, we describe how to select for a population of uniformly-expressing cells required for fluorescence experiments with the desired reporters through blasticidin drug selection or fluorescence-activated cell sorting (FACS).

### 2.1. Materials

1. HEK 293T human embryonic kidney cells (293T, ATCC^®^ CRL-3216™)
2. MDA-MB-231 metastatic breast cancer cells (231, ATCC^®^ HTB-26™)
3. HS-5 immortalized human bone marrow cell line (HS-5, ATCC^®^ CRL-11882™)
4. HS-27a immortalized human bone marrow cell line (HS-27a, ATCC^®^ CRL-2496™)
5. Cell culture media supplies
  - Dulbecco’s Modified Eagle Medium containing high glucose and pyruvate (DMEM, Gibco^®^ cat. # 11995-065)
  - Standard Fetal Bovine Serum (FBS, HyClone™ cat. # SH300088.03)
  - Penicillin-Streptomycin, 100× (P/S, Gibco^®^ cat. # 15140-122)
  - GlutaMAX Supplement™, 100× (GMx, Gibco^®^ cat. # 35050-061)
6. 0.25% Trypsin-EDTA, 1× (Gibco^®^ cat. #25200-056)
7. Sterile Phosphate Buffered Saline pH 7.4, 1× (PBS, Gibco^®^ cat. # 10010-049)
8. Various cell culture supplies such as incubators, plasticware, and sterile pipettes
9. pcDNA™6/V5-His A, B, & C Mammalian Expression Vectors (pcDNA, Invitrogen™ cat. # V220-20)
10. PCR primers for blasticidin reading frame amplification (IDT^®^ or similar vendor)
  - 5′-GTGGTTTTCCTTTGAAAAACACGATGATAATATGGCCAAGCCTTTGTCTC-3′
  - 5′-CCAGACGCGTTCAATTAATTAGCCCTCCCACACATAACCAG-3′
11. Lentiviral vector pLVX IRES puromycin (Clontech cat. # 632183)
12. Fluorescent protein LSS-mOrange (gift of V. Verkhusha, Albert Einstein College of Medicine) [8]
13. PCR primers for LSS-mOrange amplification (IDT^®^ or similar vendor)
  - 5′-ATGCGCTAGCGCCACCATGGTGAGCAAGGGCGAGGAG-3′
  - 5′-GCATGCGGCCGCTTACTTGTACAGCTCGTCCATGCCGC-3′
14. Blasticidin S HCl, powder (ThermoFisher Scientific cat. # R21001)
15. Sterile water (sterilized by institutional autoclave standards)
16. Fluorescent protein LSS-mOrange-DEVD-mKate2 (gift of V. Verkhusha, Albert Einstein College of Medicine) [8]
17. PB-CMV-MCS-EF1-Puro cDNA Cloning and Expression Vector (Systems Bioscience cat. # PB510B-1)
18. Super PiggyBac Transposase expression vector (Systems Bioscience cat. # PB210PA-1)
19. α-tri-Calcium phosphate (Sigma-Aldrich^®^ cat. # 50553)
20. FuGENE^®^ 6 Transfection Reagent (Promega cat. # E2691)
21. Enzymes, buffers, and equipment for PCR
22. Enzymes for DNA restriction digests and ligations

### 2.2. Methods

1. Culture cell lines as recommended by the supplier. We maintained the discussed cell lines in DMEM supplemented with 10% FBS, 1% P/S, and 1% GMx in a 5% CO_2_ incubator at 37 °C. We passaged these cells as necessary by trypsinization and resuspension approximately every three days.
2. For details and general protocols on transferring reporters of interest to lentiviral vectors, transfecting and transducing cell lines, and selecting for a homogenous population of stably-expressing cells, please refer to standard molecular biology texts
3. LSS-mOrange lentiviral vector generation
  - Using the PCR primers for blasticidin reading frame amplification listed in the materials, amplify the blasticidin reading frame within the pcDNA™6/V5-His A expression vector to create a selection marker for blasticidin resistance.
  - Ligate the generated PCR product (blasticidin resistance) into the BmgB1 and MluI sites on the lentiviral pLVX-IRES-puro vector to remove the gene cassette for puromycin and create the pLVX-IRES-blasticidin vector.
  - Amplify the LSS-mOrange cassette using the primers for PCR amplification of LSS-mOrange (materials section #13). Digest the resultant PCR product and ligate into the pLVX-IRES-blasticidin vector, generated in the previous step, at the NheI and NotI sites.
  - Produce lentivirus of the LSS-mOrange pLVX-IRES-blasticidin vector by transient transfection of HEK 293T cells.
  - Generate stably-expressing cell lines with the above-generated LSS-mOrange pLVX-IRES-blasticidin lentivirus and use blasticidin treatment to select for stably-expressing cells.
4. LSS-mOrange-DEVD-mKate2 vector generation
  - Excise the LSS-mOrange-DEVD-mKate2 FRET reporter cassette at the NheI and NotI sites. Ligate the cassette into the PiggyBac transposon vector at the corresponding NheI and NotI sites. A transposon must be used while generating cells expressing the LSS-mOrange-DEVD-mKate2 fluorescent reporter to prevent homologous recombination of LSS-mOrange and mKate2. These two fluorescent proteins have similar DNA sequences and that may lead to homologous recombination and result in a lack of a functional reporter.
  - Based on protocols optimized for each cell type, use calcium phosphate or FuGENE^®^ 6 to transfect 293T and 231 cells with the PiggyBac LSS-mOrange-DEVD-mKate2 transposon vector and corresponding transposase plasmid, respectively.
  - Sort for the top 30% of cells with positive expression of the LSS-mOrange-DEVD-mKate2 reporter using fluorescence-activated cell sorting (FACS). We sorted on mKate2 using a 561 nm excitation wavelength. We sorted for the top 30% to standardize the reporter expression levels.

## 3. Protocol 2: Apoptosis Imaging Experimental Setup

In this protocol, we describe experimental setup of cells utilizing the caspase-3 fluorescent reporter to study cellular apoptotic states in a variety of settings. We describe techniques, such as flat-culture, spheroids, and murine models in which cellular states are perturbed by various drugs, nutrient deprivation, and a plasmid encoding pro-apoptotic protein BAX.

### 3.1. Materials

1. Reporter verification studies
  - Plasmid encoding apoptosis regulator bcl-2-like protein 4 (BAX, gift of S. Galbán, University of Michigan)
  - pLenti-GIII-EF1a empty plasmid vector for vector control (gift of S. Galbán, University of Michigan)
2. Two-dimensional cancer models and FLIM studies
  - Trametinib (GSK112021, SelleckChem © cat. # S2673)
  - Staurosporine (Cell Signaling Technology^®^ cat. # 9953S)
  - Dimethyl Sulfoxide (DMSO, Corning^®^ cat. # 25-950-CQC)
  - Dulbecco’s Modified Eagle Medium without glucose, glutamine, or phenol red (Gibco^®^ cat. #A1443001)
  - l-Glutamine, 200 nM (Gibco^®^ cat. #25030081)
  - Glucose Solution (Gibco^®^ cat. #A2494001)
3. Culture media supplies
  - Dulbecco’s Modified Eagle Medium with high glucose and no phenol red (PRF DMEM, Gibco^®^ cat. # 31053-028)
  - Standard Fetal Bovine Serum (FBS, HyClone™ cat. # SH300088.03)
  - Penicillin-Streptomycin-Glutamine, 100× (P/S/G, Gibco^®^ cat. # 10378-016)
  - Sodium pyruvate, 100× (Gibco^®^ cat. # 11360-070)
  - Sodium dichloroacetate (DCA, Sigma-Aldrich^®^ cat. # 347795)
4. Spheroid model treatment and imaging studies
  - 384-well low volume black round bottom polystyrene NBS™ microplate, nonsterile (Corning^®^ cat. # 3676)
  - Transfer, imaging, and analysis (TRIM) plate. Note: We previously designed and described the TRIM plate to transfer and stabilize spheroids for fluorescence microscopy [13].
5. Animal models and intravital microscopy
  - 12–14-week-old female NOD.Cg-Prkdc^scid^ Il2rg^tm1Wjl^/SzJ mice (NSG, The Jackson Laboratory).
  - Small animal shaver (Wahl compact cordless trimmer or similar instrument)
  - A depilatory solution such as Nair™
  - Isoflurane
  - Various desired surgical supplies
  - Sterile 0.9% *w*/*v* NaCl solution
  - Trametinib (GSK112021, SelleckChem © cat. # S2673)
  - Dimethyl Sulfoxide (DMSO, Corning^®^ cat. # 25-950-CQC)
  - Carboxymethylcellulose, Sodium Salt, Low Viscosity (Calbiochem cat. # 217277)
  - Tween^®^ 80 (Sigma-Aldrich^®^ cat. # P4780-100ML)
6. Histology
  - Cleaved Caspase-3 (Asp175) Antibody (Cell Signaling Technology^®^ cat. #9661)

### 3.2. Methods

1. Reporter verification studies
  - For BAX apoptosis studies, transiently transfect stably-expressing 293T LSS-mOrange-DEVD-mKate2 or stably-expressing 293T LSS-mOrange cells using increasing concentrations of either the BAX-encoding plasmid or empty vector (control) and calcium phosphate. Amounts of 500, 1000, and 2000 ng plasmid proved a useful range for our study.
  - Wait 24 h after transfection and seed 1.4e5 cells of BAX-293T LSS-mOrange-DEVD-mKate2 or BAX-293T LSS-mOrange cells into the 6 wells of an entire 6-well plate using phenol red free (PRF) DMEM supplemented with 10% FBS and 1% P/S/G. Using phenol red-free media for fluorescence imaging assays minimizes background noise.
  - 24 h after re-seeding the BAX-transfected cells, image cells for caspase-3 LSS-mOrange-DEVD-mKate2 reporter activation and compare to LSS-mOrange control cell lifetimes for reporter verification using FLIM.
2. Two-dimensional cancer models and FLIM studies
  - For studying effects of nutrient deprivation and various compounds (DCA, trametinib, and staurosporine) on apoptosis and fluorescence lifetime, seed 1.4e5 231 LSS-mOrange-DEVD-mKate2 cells into each well of an entire 6-well plate using PRF DMEM supplemented with 10% FBS and 1% P/S/G.
  - Treat cells 24 h before performing imaging studies.
    - Nutrient deprivation: Treat cells with phenol red, glutamine, and glucose-free DMEM supplemented with 10% FBS and either 1% glucose, 1% glutamine, or neither.
    - DCA: Treat cells with 40mM DCA dissolved in phenol red, glutamine, and glucose-free DMEM supplemented with 10% FBS and 1% sodium pyruvate and either 1% glucose or 1% glutamine.
    - Compound studies: Treat cells with 100 nM trametinib, 1 μM staurosporine, or DMSO vehicle control in PRF DMEM supplemented with 10% FBS and 1% P/S/G.
  - Image cells for caspase-3 LSS-mOrange-DEVD-mKate2 reporter activation and changes in LSS-mOrange fluorescence lifetime with nutrient deprivation and compound treatment.
3. Cancer spheroid model and FLIM studies
  - Prepare for seeding cells for spheroid formation by sterilizing 384-well plates by radiating with UV light for 90 s. Formulate media with base PRF DMEM supplemented with 10% FBS and 1% P/S/G.
  - Place 3000 total cells in 25 μL of media per well with 20% cancer cells (LSS-mOrange-DEVD-mKate2 expressing 231 cells) mixed with 80% bone marrow stromal cells (either HS-5 or HS-27A cells). Seeding cancer cells with stromal cells allow for the cells to form viable spheroids that some cancer cell lines may not be able to form alone.
  - After waiting 24 h for cells to form compact spheroids, gently remove 18 μL of media from each spheroid-containing well and add 20 μL fresh media containing either 100 nM trametinib or DMSO as its vehicle control.
  - 24 h after treatment, transfer spheroids to a TRIM plate and image for 3D caspase-3 reporter activation using FLIM imaging.
4. Murine models and intravital tumor microscopy
  - Verify that animal procedures have been approved by the local IACUC.
  - Create a concentrated suspension of 5e5 LSS-mOrange-DEVD-mKate2 expressing 231 cells/50 μL in sterile NaCl solution. Keep the solution cold by leaving cell suspension on ice until use.
  - Prepare 12- to 14-week-old female NSG mice for surgery and inject 50 μL of the above-generated cell suspension into both fourth inguinal mammary fat pads as we have previously described [14]. We choose to use NSG mice as they are highly immunocompromised and allow for effective growth of injected human breast cancer cells.
  - Once 3 to 4 mm diameter tumors have formed, treat mice daily with of either 1 mg/kg trametinib or appropriate vehicle control by 50 μL of oral gavage as previously detailed [15]. Using the aforementioned parameters, tumor formation requires approximately 20 days. This length of time varies with mouse strain, cancer cell type, and concentration of injected cells.
  - Using the FLIM imaging techniques described in Protocol 4, perform intravital microscopy for caspase-3 activation and LSS-mOrange lifetime after both 8 and 14 days of consecutive treatment.
5. Histological Verification of in-vivo FLIM studies
  - Following 14 days of either treatment or vehicle control, remove the formed mammary fat pad tumors and fix using standard techniques and 10% formalin. Post-fixation, follow standard protocols and embed, slice, and process tissue.
  - Perform histology with hematoxylin and eosin staining and immunohistochemistry for caspase-3 reporter cleavage with the Cleaved Caspase-3 (Asp175) Antibody. Please refer to standard texts for details on immunohistochemistry and staining protocols.
  - Image slides and determine the fraction of cells positive for caspase-3 reporter cleavage. Use these data to verify results from the acquired FLIM data.

## 4. Protocol 3: Glycolysis Stress Tests

In Protocol 3, we describe how to use the commercially available Seahorse Bioscience XFe analyzer and glycolysis stress test. The metabolic flux data are correlated with apoptosis measurements from the caspase-3 reporter under various nutrient and drug conditions. 

### 4.1. Materials

1. Culture media supplies
  - Dulbecco’s Modified Eagle Medium with high glucose and pyruvate (DMEM, Gibco^®^ cat. # 11995-065)
  - Standard Fetal Bovine Serum (FBS, HyClone™ cat. # SH300088.03)
  - Penicillin-Streptomycin, 100× (P/S, Gibco^®^ cat. # 15140-122)
  - Glutamine, 100× (Gibco^®^ cat. # 25030-081
  - Pyruvate, 100× (Gibco^®^ cat. # 11360-070)
  - Glucose, dissolved in sterile water, 1 M (Sigma Aldrich, cat. # G5767)
  - Miscellaneous desired cell culture supplies such as plasticware, incubators, and sterile pipettes
2. Non-CO_2_, 37 °C incubator
3. Seahorse Bioscience XF96 96-well cell culture microplate (Agilent, Santa Clara, CA, USA, part # 101085-004)
4. Seahorse Bioscience XF^e^96 extracellular flux assay kit (Agilent, Santa Clara, CA, USA, part # W26316) for the XF^e^96 analyzer
5. Seahorse Bioscience XF Base Medium (part # 102353-100)
6. Seahorse Bioscience XF Calibrant (Agilent, Santa Clara, CA, USA, part # 100840-000)
7. Seahorse Bioscience Glycolysis Stress Test kit (part # 103017-100)

### 4.2. Methods

1. 24 h before performing the metabolic flux assay, seed 5e3 231 cells in 80 μL DMEM supplemented with 10% FBS, 1% P/S, and 1% glutamine per well in a Seahorse Bioscience XF96 96-well plate. Add 200 μL XF calibrant to each well of the utility plate. Incubate cells in a 37 °C, 5% CO_2_ incubator and utility plate in a 37 °C, non-CO_2_ incubator overnight.
2. One day after seeding cells, prepare Seahorse assay medium. Mix the below-listed ingredients, adjust the solution’s pH to 7.4, and sterile filter it. Store the medium at 37 °C until use.
  - 100 mL Seahorse Bioscience XF Base medium pre-warmed to 37 °C
  - 1 mL of 100 mM pyruvate
  - 1 mL of 200 mM glutamine
  - 1 mL of 1 M glucose solution
3. Change the medium in the cell culture plate by gently washing each well twice with 180 μL of the Seahorse assay medium. In each well, gently add the final volume of 180 μL of the Seahorse assay medium. Before inserting the cell culture plate into the XF analyzer, place the plate into a non-CO_2_ incubator at 37 °C for 1 h.
4. Following the instructions found in the Seahorse manual, suspend the components from the glycolysis stress test (glucose, oligomycin, and 2-deoxyglucose) in the Seahorse assay medium.
5. Using a multichannel pipette, inject 10 mM glucose, 2 mM oligomycin, and 100 mM 2-deoxyglucose to Ports A, B, and C of the utility plate, respectively, immediately before beginning the experiment.
6. Following the instructions displayed on the analyzer, place the utility plate inside the XF analyzer. Subsequently, insert the prepared cell culture plate.
7. Setup the XF analyzer by indicating the injection and measurement strategies. Follow subsequent instructions from the software to perform the experiment.
8. Using the Wave program provided by Seahorse Bioscience, analyze the acquired data.

## 5. Protocol 4: FLIM Techniques and Analysis

Here, we outline the steps for imaging the caspase-3 reporter using FLIM in the various experimental environments discussed in Protocol 2. FLIM measures the lifetime of LSS-mOrange in the reporter, identifying cells undergoing apoptosis by a longer lifetime of this fluorescent protein. 

### 5.1. Materials

1. 2-photon upright imaging system with variable laser power and compatible 25× objective (Olympus FVE1000 MPE microscope and 25× NIR corrected objective, XLPLN25XWMP, NA = 1.05, Olympus, Tokyo, Japan or similar product)
2. 80 MHz pulsed scanning laser (Spectra-Physics Mai Tai-Deep Sea or comparable product)
3. 572/15 nm bandpass emission filter
4. FLIM instrument with frequency domain analysis capabilities (FastFLIM, ISS, Champaign, IL, USA or comparable product)
5. Vista Vision Software or similar product for data analysis (ISS, Champaign, IL, USA)

### 5.2. Methods

1. Allow laser to stabilize at 820 nm by turning it on for at least 45 min before calibration. Perform FLIM system lifetime calibration as per the manufacturer’s instructions. We used a 4.1 ns fluorescein slide for lifetime calibration.
2. Immerse objective in the sample (6-well plate, TRIM plate, or tumor) and focus on a region of interest. Follow protocols to image orthotopic mammary tumors as we have previously discussed [16].
3. Using an 820 nm wavelength, excite the LSS-mOrange fluorescent protein. We found a 256 × 256-pixel image with a 100 μs dwell time over 15–30 frames allow for sufficient counts and accurate data.
4. LSS-mOrange is a long stokes shift fluorescent protein and emits in the orange channel (572 nm). Acquire and measure fluorescence lifetime of molecules in the orange channel. Literature reports the known LSS-mOrange lifetime as 2.75 ± 0.07 ns [17].
5. After imaging the samples of interest, open and analyze images with ISS VistaVision or similar processing software. Using the Multi-Image Phasor Analysis function, set the minimum threshold count to eliminate background data and the Gaussian smoothing operation to 3 to smooth acquired data while maintaining spatial accuracy.
6. Use the phasor plot and ROI function to define the portion of pixels with an increased LSS-mOrange lifetime. Pseudocolor this population as red and the shorter lifetime pixels as yellow with another ROI. Cells displaying increased amounts of yellow or red indicate increased or decreased amounts of apoptosis, respectively. Detailed reports on fluorescence lifetime analysis using phasor plots have been previously published [18].

## 6. Results

**Figure 1:**
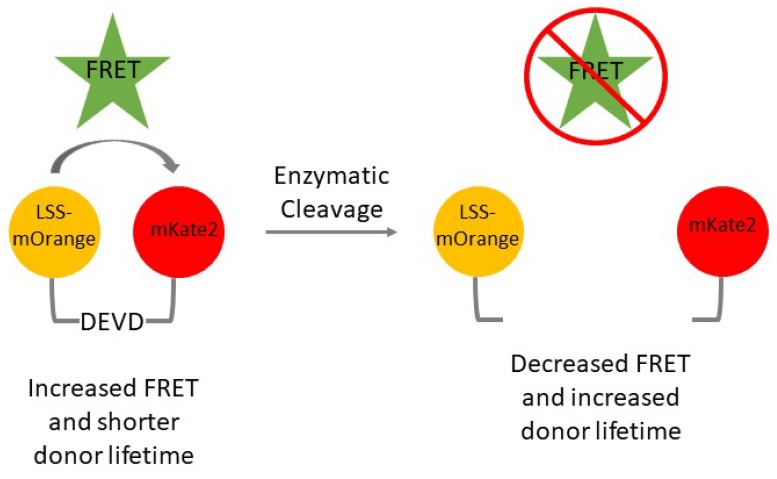
Schematic representation of the LSS-mOrange-DEVD-mKate2 reporter. The intact DEVD linker between LSS-mOrange and mKate2 causes the two fluorescent proteins to be spatially close and experience increased FRET. This increase in FRET causes a decrease in the donor (LSS-mOrange) lifetime. Once the DEVD linker is enzymatically cleaved by active caspase-3, LSS-mOrange and mKate2 can spatially separate. This causes FRET to decrease and the donor (LSS-mOrange) lifetime to increase.

FRET between LSS-mOrange and mKate2 will cause the higher-energy donor fluorescent protein (LSS-mOrange) to have a shorter fluorescence lifetime. Energy transfer between these two molecules will occur if they are spatially close to one another, as shown in the intact reporter construct. Once the DEVD sequence has been cleaved by caspase-3 during apoptosis, the LSS-mOrange and mKate2 FRET pair spatially separate. Separation of these proteins minimizes energy transfer from LSS-mOrange to mKate-2 and increases the donor’s lifetime (Figure 1). 

**Figure 2:**
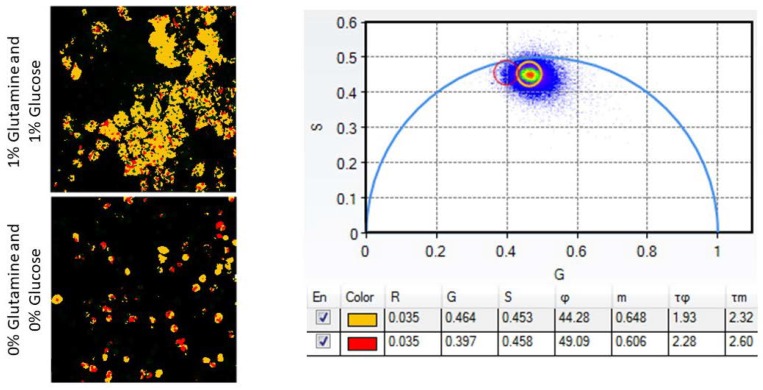
MDA-MB-231 cells show increased apoptosis with nutrient starvation. Left. MDA-MB-231 cells that have been starved of both glutamine and glucose (**bottom**) show a higher proportion of red pixels (indicating apoptosis) than the control, nutrient-rich condition (**top**). **Right**. The phasor plot indicates the ROIs that were selected. The numbers below the phasor plot indicate ROI spatial information. Points nearest the origin on the *x*-axis have a longer lifetime. The orange ROI indicates pixels with a shorter lifetime (no caspase-3 activity, FRET is occurring) and the red ROI indicates pixels with a longer lifetime (caspase-3 activity and apoptosis, no FRET).

In Protocols 2 and 4, we discuss multiple experimental setups and imaging protocols, respectively. We expect the LSS-mOrange control cells to display a longer fluorescence lifetime than cells expressing fused LSS-mOrange-DEVD-mKate2 due to the lack of energy being transferred from the donor protein. Any treatment that causes apoptotic cell death will display an increased fraction of cells with a longer lifetime of LSS-mOrange due to cleavage at DEVD sites. The shift to the longer lifetime of LSS-mOrange occurs in 2D environments with increasing amounts of BAX-encoding plasmid, decreasing the availability of essential nutrients, and treating with different concentrations of pro-apoptotic drugs (Figure 2). The 3D spheroid and murine models will also display the same patterns; groups treated with trametinib will display increased apoptosis over their vehicle control.

Protocol 3 details how to perform a metabolic flux assay on cells used in the FLIM studies. We used the commercially available metabolic flux assay and glucose stress test along with the Seahorse Bioscience XFe analyzer. These data can be correlated with a 2D nutrient deprivation study and subsequent FLIM imaging of the LSS-mOrange-DEVD-mKate2 reporter. Metabolically active cells with increased rates of both oxygen consumption and extracellular acidification will display less apoptotic cell death.

Future implications of this technology include screening for compounds that induce cellular apoptosis in cancer cells. While cells in 2D environments may present with morphological changes when beginning to undergo apoptosis, it is more difficult to visualize these changes in cells in 3D environments. The usefulness of FRET apoptosis reporter constructs is highlighted by the recent use of a similar construct to detect caspase-3 activation in a mouse xenograft model [19]. This reporter allows for efficient screening of apoptosis in complex environments and we have previously used this reporter to quantify apoptosis in 3D and mouse models of breast cancer [9].
